# Supplementary material for: Estimating CO2 emissions due to present and future suborbital space tourism industry
Source: PLoS One. 2025 Aug 20;20(8):e0328456. doi: 10.1371/journal.pone.0328456 (PMC12367173; doi:10.1371/journal.pone.0328456)
Supplement: Attachment — Submitted filename: responses to review comments.pdf [file pone.0328456.s003.pdf]

Responses to Review Comments

(Manuscript No.: PONE-D-25-00871)

Angela M. Huang and Yangyang Xu*

(* corresponding author)

The authors would like to thank the editor and reviewers for their careful review and valuable comments/suggestions. Below please find our point-by-point responses to the editor's and reviewers' comments and the changes we made to address the comments/suggestions. All the review comments/suggestions are presented in ***bold Italic Arial*** while our responses are presented in Normal Arial. **All the page numbers or line numbers cited herein are based on the version of the paper with tracked changes, which is submitted in this revision.**

Editor's comments:

When submitting your revision, we need you to address these additional requirements.

1. Please ensure that your manuscript meets PLOS ONE's style requirements, including those for file naming. The PLOS ONE style templates can be found at

https://journals.plos.org/plosone/s/file?id=wjVg/PLOSOne_formatting_sample_main_body.pdf and

https://journals.plos.org/plosone/s/file?id=ba62/PLOSOne_formatting_sample_title_authors_affiliations.pdf

Response: We have carefully checked the journal requirements for manuscript, title, authors and affiliations, and have made appropriate changes to ensure our manuscript being in compliance with the requirements.

2. We note that your Data Availability Statement is currently as follows: "All relevant data are within the manuscript and in Supporting Information files."

Please confirm at this time whether or not your submission contains all raw data required to replicate the results of your study. Authors must share the "minimal data set" for their submission. PLOS defines the minimal data set to consist of the data required to replicate all study findings reported in the article, as well as related metadata and methods (https://journals.plos.org/plosone/s/data-availability#loc-minimal-data-set-definition).

For example, authors should submit the following data:

- The values behind the means, standard deviations and other measures reported;

- The values used to build graphs;

- The points extracted from images for analysis.

Authors do not need to submit their entire data set if only a portion of the data was used in the reported study.

If your submission does not contain these data, please either upload them as Supporting Information files or deposit them to a stable, public repository and provide us with the relevant URLs, DOIs, or accession numbers. For a list of recommended repositories, please see https://journals.plos.org/plosone/s/recommended-repositories.

If there are ethical or legal restrictions on sharing a de-identified data set, please explain them in detail (e.g., data contain potentially sensitive information, data are owned by a third-party organization, etc.) and who has imposed them (e.g., an ethics committee). Please also provide contact information for a data access committee, ethics committee, or other institutional body to which data requests may be sent. If data are owned by a third party, please indicate how others may request data access.

Response: Our collected data are included in the tables in the manuscript. Most of our figures are generated based on the mathematical formulas derived in this study. We

submit an excel spreadsheet to include all the numbers that are calculated from the formulas in this submission.

3. We note that Figure 1 in your submission contain copyrighted images. All PLOS content is published under the Creative Commons Attribution License (CC BY 4.0), which means that the manuscript, images, and Supporting Information files will be freely available online, and any third party is permitted to access, download, copy, distribute, and use these materials in any way, even commercially, with proper attribution. For more information, see our copyright guidelines: http://journals.plos.org/plosone/s/licenses-and-copyright.

We require you to either (a) present written permission from the copyright holder to publish these figures specifically under the CC BY 4.0 license, or (b) remove the figures from your submission:

a. You may seek permission from the original copyright holder of Figure 1 to publish the content specifically under the CC BY 4.0 license.

We recommend that you contact the original copyright holder with the Content Permission Form (http://journals.plos.org/plosone/s/file?id=7c09/content-permission-form.pdf) and the following text:

"I request permission for the open-access journal PLOS ONE to publish XXX under the Creative Commons Attribution License (CCAL) CC BY 4.0 (http://creativecommons.org/licenses/by/4.0/). Please be aware that this license allows unrestricted use and distribution, even commercially, by third parties. Please reply and provide explicit written permission to publish XXX under a CC BY license and complete the attached form."

Please upload the completed Content Permission Form or other proof of granted permissions as an "Other" file with your submission.

In the figure caption of the copyrighted figure, please include the following text: "Reprinted from [ref] under a CC BY license, with permission from [name of publisher], original copyright [original copyright year]."

b. If you are unable to obtain permission from the original copyright holder to publish these figures under the CC BY 4.0 license or if the copyright holder's requirements are incompatible with the CC BY 4.0 license, please either i) remove the figure or ii) supply a replacement figure that complies with the CC BY 4.0 license. Please check copyright information on all replacement figures and update the figure caption with source information. If applicable, please specify in the figure caption text when a figure is similar but not identical to the original image and is therefore for illustrative purposes only.

Response: We have re-created Figure 1 to avoid copyright issues and added more information about the space and space tourism market. Now all the information in Figure 1 is from the public domain.

4. We note you have included a table to which you do not refer in the text of your manuscript. Please ensure that you refer to Table 3 in your text; if accepted, production will need this reference to link the reader to the Table.

Response: Table 3 was referred to in the text, which is in Line 393. The sentence in the paper is cited below for an easy assessment.

Line 393
analytical framework (Eqns. 6, 14, and 16). The reported dry masses of the spacecraft of Blue Origin, Virgin Galactic, and SpaceX are listed in Table 3. The dry mass of the Virgin Galactic

Reviewers' comments:

Comments to the Author

1. Is the manuscript technically sound, and do the data support the conclusions?

The manuscript must describe a technically sound piece of scientific research with data that supports the conclusions. Experiments must have been conducted rigorously, with appropriate controls, replication, and sample sizes. The conclusions must be drawn appropriately based on the data presented.

Reviewer #1: Partly

Reviewer #2: Partly

Response: We have conducted additional analysis as suggested by the reviewer(s) and additional statements have been added to the conclusion.

2. Has the statistical analysis been performed appropriately and rigorously?

Reviewer #1: I Don't Know

Reviewer #2: Yes

Response: We have conducted Monto Carlo analysis to assess the variation of CO_2 emissions considering the possible variations of ***TSFCs***, and ***emissions factors***. The new data are included in this revised manuscript.

3. Have the authors made all data underlying the findings in their manuscript fully available?

The PLOS Data policy requires authors to make all data underlying the findings described in their manuscript fully available without restriction, with rare exception (please refer to the Data Availability Statement in the manuscript PDF file). The data

should be provided as part of the manuscript or its supporting information, or deposited to a public repository. For example, in addition to summary statistics, the data points behind means, medians and variance measures should be available. If there are restrictions on publicly sharing data—e.g. participant privacy or use of data from a third party—those must be specified.

Reviewer #1: Yes

Reviewer #2: Yes

Response: We appreciate the reviewers' confirmation.

4. Is the manuscript presented in an intelligible fashion and written in standard English?

PLOS ONE does not copyedit accepted manuscripts, so the language in submitted articles must be clear, correct, and unambiguous. Any typographical or grammatical errors should be corrected at revision, so please note any specific errors here.

Reviewer #1: Yes

Reviewer #2: No

Response: We have proofread the manuscript and made various changes throughout the paper to remove typos and improve wording and sentencing. Our corrections are indicated by the tracked changes.

<u>Reviewer #1</u>:

While the article presents an interesting and relevant topic regarding CO_2 emissions associated with suborbital flights, there are several aspects that raise concerns about its suitability for publication in this prestigious journal. Firstly, the paper's approach seems somewhat elementary and may lack the depth expected for a high-impact journal. The analysis is based on basic physical principles and fundamental kinetics of launching and landing, which, although informative, may not offer the level of novelty or advanced methodology required to stand out in the field.

The models proposed, while valuable, appear to be quite basic and do not seem to introduce any groundbreaking insights or advancements in the methodology used for CO_2 estimation.

Response: We appreciate the comment on our methodology and would like to share our perspective as follows.

Our study is based on the well-established Newton's law and aerodynamics principles, which have been the basic approach to analyze spacecraft kinetics for decades. The simplicity of this approach is an advantage in terms of transferability and generalization as new spacecrafts (weights, shapes, fuel) are developed. This is the motivation for our approach to develop an analytical framework that is based on the first principles and reliable data to provide a reasonable estimate of the emissions from suborbital travel.

This simpler approach is preferred as detailed configurations of these spacecrafts are not public information. Indeed, space tourism is an emerging sector and there are very limited studies published on it, and the very few published estimations of emissions from suborbital travel have significant discrepancy that led to significant dispute and tension, which is discussed in our paper in the Introduction between Lines 95-109, as cited below.

Lines 95 - 109

types, fuel efficiency, engines, flight characteristics, etc. Very few~~limited~~ studies on suborbital GHG emissions have relied on indirect information, leading to significant~~large~~ uncertainty and potential disputes. For example, Virgin Galactic claimed that its carbon footprint per passenger was comparable~~similar~~ to a business-class round trip from London to New York; however, the Financial Times reported that it emitted~~released~~ 1,238 kg of CO_2 per passenger (42) ~~,~~ 60 times higher. Fawkes (43) provided an even higher estimate for Virgin Galactic's SpaceShipTwo at 3,113 kg per passenger. Even greater~~more~~ disputes arise over the ~~on~~ estimates of non-traditional aviation fuels, such as liquid hydrogen. The World Inequality Report argued that an 11-minute suborbital joyride by Blue Origin created more carbon emissions than 1 billion people produced in their entire lifetime (44). The claim received widespread~~comprehensive~~ media~~news~~ coverage but was questioned in terms of~~regarding~~ how the carbon footprint of ~~they had estimated~~ liquid hydrogen was estimated~~'s carbon footprint~~. Rigorous and independent studies should be conducted~~performed~~ to quantify CO_2 emissions associated with suborbital travel and ~~also~~ the indirect carbon footprint of fuel production (43). The latter is particularly relevant to hydrogen as it does not directly emit~~release~~ CO_2 when burned.

The discrepancy is attributable to the fact that there is no well-established, sound framework to analyze the flight of suborbital spacecraft, so the existing studies based their estimations on analysis tools and data from aviation, which resulted different results. The inconsistency, if not addressed thoroughly, can seriously delay our action to mitigate the emission issue from this emerging sector.

Therefore, the merit of our paper lies in three perspectives:

- It develops a simple and sound analytical framework based on first principles with variable and flexible parameter choice to analyze single-stage, vertically launched rockets, which fills the gap of existing studies and can be used for many other applications,
- It links a developed analytical framework with spacecraft-specific and fuel-specific parameters to accurately estimate fuel consumptions and CO_2 emissions, and

- It provides comprehensive emission data for different rocket fuels, which are not available anywhere else in the public domain.

We agree with the reviewer that the paper can be further improved in terms of analysis, interpretation of the findings and broad implications. Therefore, we have done the following to address the reviewer's concern:

- Performed a detailed Monte Carlo simulation to account for the uncertainty of key parameters, which can be found from Lines 509 to 521, as cited below. The results have been added to Figures 4-7.

Lines 509 - 521
**Monte Carlo analysis**<br><br>The calculation of emissions involves two parameters – fuel consumptions and emission factors. Since the TSFC of the fuels varies within a range (listed in Table 5), the calculated fuel consumption will fall into a range. The emissions factors (listed in Table 6) also fall into a range. Therefore, a Monte Carlo simulation, which is a computational technique that uses repeated random sampling to obtain numerical results, is utilized to estimate the emissions considering the combination of various scenarios of TSFCs and emission factors. Both TSFCs and emission factors are assumed to fall into a normal distribution, and the lower and upper bounds of the distributions are assumed to correspond to 10% and 90% percentile, respectively. To ensure accuracy and convergence, the simulation starts with 2,000 cycles and is increased by 500 cycles each time until the yielded mean and standard deviation become nearly unchanged. The Monte Carlo simulation is used to quantify not only CO_2 emissions from fuel combustion or production but also the total CO_2 emissions, i.e., emissions from combustion + production.

Figure 4c is cited below to illustrate the changes we made, and the changes in other figures can be found in the revised paper.

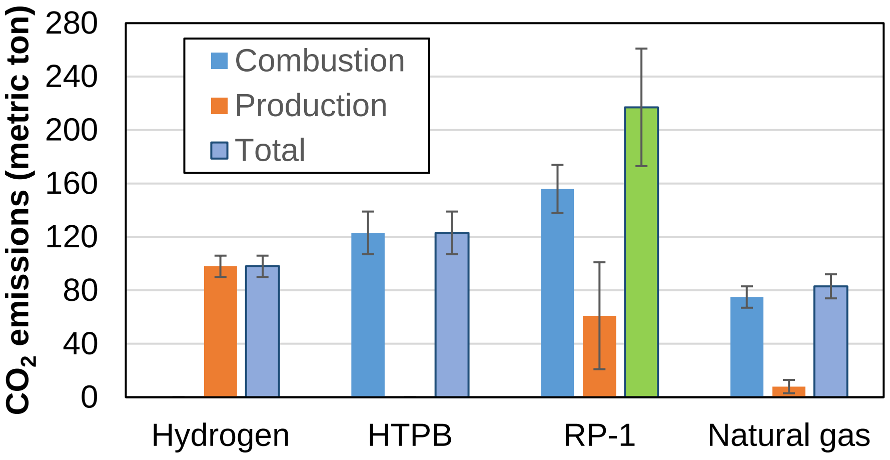

- Provided more insight into the implications of the results of this paper, such as the long-term emission potential, which can be found from Lines 729 to 745 as cited below.

Lines 729 - 745
The long-term implications of CO_2 emissions from suborbital tourism are broad and impactful, considering that it is still at the early stage of adoption. We even consider that 85,000 launches per year in 2030s would be a small portion when the market becomes mature. Logistic function has been widely used by market researchers to predict the public acceptance of newly emerging technology, which has successfully predicted the market penetration of cell phones, personal computers, smart phones, electrical vehicles, etc. (124). A genetic logistic function is divided into three phases based on its market penetration: early adoption (<10% market penetration), wide acceptance (10%-90% market penetration), and maturation (>90% market penetration). In the early adoption phase, the sales growth is slow, while in the wide acceptance phase the growth is rapid and exponential (125). Considering that 85,000 flights are still significantly less than 10% of total addressable market (TAM) of suborbital tourism, we can infer that suborbital tourism will be still at the early adoption phase. Smart phones took about 7 years to enter the second phase, while personal computers took slightly longer than 10 years. Thus, using that analogy suborbital tourism may enter the wide acceptance phase after mid-2030, and by then the annual launches could be a few times more than 85,000 flights, which can really impose a challenge due to its possible CO_2 emissions. More detailed analysis could be possible in the future when more market data on suborbital tourism becomes available.

- Discussed the future work, which can be found from Lines 788 to 790.

Lines 788 - 790
compared with others, it has little potential for future reduction. Also of note is that our estimate for Virgin Galactic does not include ~~the indirect~~indirect emissions from fuel production; therefore, the estimation can be refined once more fuel production information becomes available. ~~SpaceX~~

Furthermore, while the results are intriguing—particularly the comparison of emissions from different spacecraft and fuels—the analysis remains at a level that may be more suited to a classroom or introductory research project rather than a comprehensive scientific study.

The study provides useful data but does not seem to fully explore or address the broader implications of these findings in the context of global environmental policy or the technological innovations needed to mitigate such emissions.

Response: Again, we generally agree that our analysis is based on basic scientific principles (rather than detailed simulation as we do not have access to these computational tools and key parameters available to the rocket companies), but the simplicity may be reviewed as an advantage, since the very few published estimations are controversial and widely questioned in the public domain. Our simple and straightforward approach can be easily assessed by readers and further improved upon once more detailed quantitative information becomes publicly available, which can help ease the existing dispute.

More importantly, a significant effort of this study is data collection including rocket fuel types, engine efficiencies, TSFCs, emission factors (combustion and production), flight characteristics, and growth potentials in the future. The data, which played a significant role in the analysis, were scattered in papers, reports, carriers' specs, etc., which took us nearly two years to gather. Thus, our paper provides a collection of relevant datasets. We believe that the analytical and data merit of this paper should be jointly considered to justify the contribution of this paper.

To signify the results of this study, we added more interpretation and discussions to put the findings into the broader context of global environmental policy as well as direction of innovation directions, as suggested.

In summary, we have made significant improvements in our studies, which include:

- Collecting more data to account for the possible variation range of emissions factors for different fuels. The related revisions are Table 6, and Lines 483 – 499 as cited below:

Lines 483 - 499

The CO_2 emissions associated with RP-1 fuel are estimated based on US EPA emission factor. The combustion of each gallon of kerosene would directly release 9.98 kg of CO_2 (100), which is converted to a mass ratio, i.e., emission factor. Koroneos, Dompros (101) and GHG Management Institute (95) indicated the emission factors associated with ~~0.051 kg/MJ for emissions associated with~~ kerosene production range from 0.5 to 2.1, which ~~is used here to quantify~~are listed in ~~the CO_2 emissions from kerosene production (~~Table 6~~)~~.

Detailed data on CO_2 emissions associated with natural gas production is not available, but Zhang, Cusworth (96) concluded that natural gas liquefaction released only 5 - 16% CO_2 relative to end-use combustion. Okamura, Furukawa and Tamura, Tanaka reported that 230 – 410 g of CO_2 is released to liquefy each kilogram of natural gas (102, 103), which is equivalent to 8.5% to 15.2% of end-use emissions. Howarth suggested that liquefaction accounts for 8.8% CO_2 emissions relative to end-use based on the U.S. data (104). Thus, it is supportive to use 5 – 16% as the lower and upper bound of CO_2 emissions due to liquefaction. ~~The average (10%) is used to estimate the CO_2 emission associated with production.~~ It is noteworthy that at this moment, SpaceX is using natural gas for its raptor engines, but Blue Origin indicated that its future BE-4 engine will use liquefied natural gas as well. That means natural gas engines may become more popular in the future.

Table 6

	Direct emissions due to Combustion	Indirect emissions due to Production	
	Current and /Future (g/g)	Current (mid-2020s) (g/g)	Future (2030s) (g/g)
**Hydrogen**	0	10.110.7 – 11.8	7.156.7 – 7.6
**HTPB**	2.2 – 2.6*	----	----
**RP-1**	3.16 - 3.5	0.5 - 2.1	0.4 - 1.54
**CH_4**	2.6 - 2.75	0.13 – 0.440.27	0.1 - 0.18
Note: *the emission factor range for HTPB is calculated from its theoretical emissions from combustion and then considering engine efficiency.			

- Conducting Monte Carlo simulations to assess the combination of different scenarios to better understand the possible emissions. The related revisions are Lines 509 – 521, as cited below:

Lines 509 - 521

Monte Carlo analysis

The calculation of emissions involves two parameters – fuel consumptions and emission factors. Since the TSFC of the fuels varies within a range (listed in Table 5), the calculated fuel consumption will fall into a range. The emissions factors (listed in Table 6) also fall into a range. Therefore, a Monte Carlo simulation, which is a computational technique that uses repeated random sampling to obtain numerical results, is utilized to estimate the emissions considering the combination of various scenarios of TSFCs and emission factors. Both TSFCs and emission factors are assumed to fall into a normal distribution, and the lower and upper bounds of the distributions are assumed to correspond to 10% and 90% percentile, respectively. To ensure accuracy and convergence, the simulation starts with 2,000 cycles and is increased by 500 cycles each time until the yielded mean and standard deviation become nearly unchanged. The Monte Carlo simulation is used to quantify not only CO_2 emissions from fuel combustion or production but also the total CO_2 emissions, i.e., emissions from combustion + production.

- Using an analogical approach to showcase the importance of emissions from suborbital flights in the future. The related revisions are in Lines 729 – 745.

Lines 729 - 745
The long-term implications of CO_2 emissions from suborbital tourism are broad and impactful, considering that it is still at the early stage of adoption. We even consider that 85,000 launches per year in 2030s would be a small portion when the market becomes mature. Logistic function has been widely used by market researchers to predict the public acceptance of newly emerging technology, which has successfully predicted the market penetration of cell phones, personal computers, smart phones, electrical vehicles, etc. (124). A genetic logistic function is divided into three phases based on its market penetration: early adoption (<10% market penetration), wide acceptance (10%-90% market penetration), and maturation (>90% market penetration). In the early adoption phase, the sales growth is slow, while in the wide acceptance phase the growth is rapid and exponential (125). Considering that 85,000 flights are still significantly less than 10% of total addressable market (TAM) of suborbital tourism, we can infer that suborbital tourism will be still at the early adoption phase. Smart phones took about 7 years to enter the second phase, while personal computers took slightly longer than 10 years. Thus, using that analogy suborbital tourism may enter the wide acceptance phase after mid-2030, and by then the annual launches could be a few times more than 85,000 flights, which can really impose a challenge due to its possible CO_2 emissions. More detailed analysis could be possible in the future when more market data on suborbital tourism becomes available.

The additional analysis has allowed us to assess the uncertainty, which is reflected by the newly added error bars in Figures 4 and 7, as shown by Figure 4c as an example to illustrate the change.

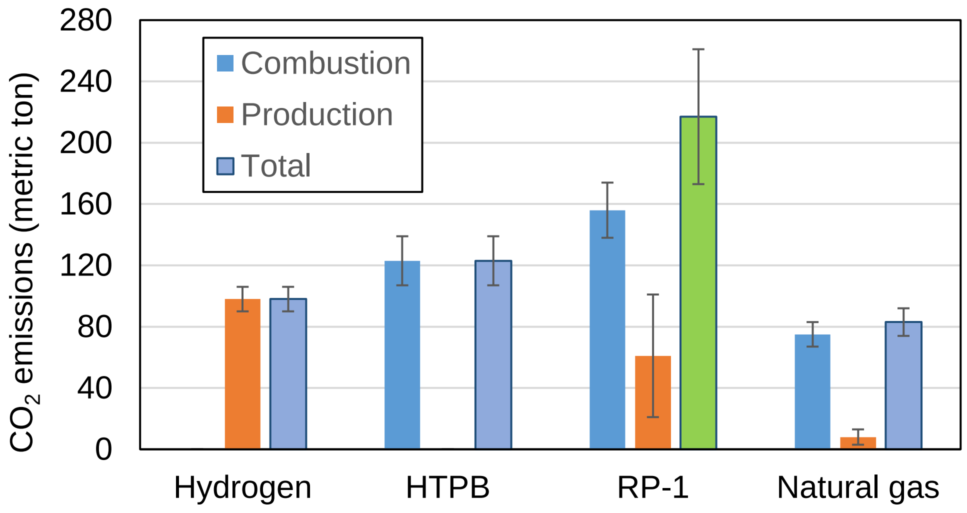

Additionally, while the potential scale of emissions from suborbital flights is highlighted, the article does not provide sufficient data or a detailed discussion on how these emissions could be reduced or managed. Given the growing concern around climate change and CO_2 emissions, this is a crucial aspect that would enhance the paper's contribution to the field.

Response: We appreciate the comment and have expanded the discussion to stress the significance of the findings and possible strategies to mitigate emissions for suborbital travels. From Lines 729 to 745, we have discussed the market potential of suborbital tourism, pointed out we are in the early adoption phase, and emphasized that it could be very challenging after mid-2030s when it enters the wide acceptance phase.

Lines 729 – 745
The long-term implications of CO_2 emissions from suborbital tourism are broad and impactful, considering that it is still at the early stage of adoption. We even consider that 85,000 launches per year in 2030s would be a small portion when the market becomes mature. Logistic function has been widely used by market researchers to predict the public acceptance of newly emerging technology, which has successfully predicted the market penetration of cell phones, personal computers, smart phones, electrical vehicles, etc. (124). A genetic logistic function is

divided into three phases based on its market penetration: early adoption (<10% market penetration), wide acceptance (10%-90% market penetration), and maturation (>90% market penetration). In the early adoption phase, the sales growth is slow, while in the wide acceptance phase the growth is rapid and exponential (125). Considering that 85,000 flights are still significantly less than 10% of total addressable market (TAM) of suborbital tourism, we can infer that suborbital tourism will be still at the early adoption phase. Smart phones took about 7 years to enter the second phase, while personal computers took slightly longer than 10 years. Thus, using that analogy suborbital tourism may enter the wide acceptance phase after mid-2030, and by then the annual launches could be a few times more than 85,000 flights, which can really impose a challenge due to its possible CO_2 emissions. More detailed analysis could be possible in the future when more market data on suborbital tourism becomes available.

From Lines 746 – 751, we have added discussions regarding the potential solutions to mitigate CO_2 emissions from this sector.

Lines 746 - 751

According to this study, the key factors impacting emissions are engine efficiency (i.e., TSFC), types of fuel (i.e., emissions factors and fuel mass), and spacecraft mass. Improving spacecraft mass and engine efficiency is challenging unless a revolutionary breakthrough occurs in the near future. The choice of fuel can significantly affect emissions, and our study shows that the long-term liquid hydrogen and natural gas are better choice than other fuels so they should be prioritized to mitigate CO_2 emissions associated with suborbital tourism.

In conclusion, while the study has potential, it would benefit from a deeper analysis, more advanced modeling, and a clearer connection to the broader implications of suborbital flight emissions. As it stands, it may not meet the high standards expected by this journal, and I would recommend reconsideration after further revisions and improvements.

Response: We appreciate that the reviewer confirmed the potential of our paper and have considered all the comments carefully.

In this revision, we have (1) clarified the advantage of our simplistic approach, (2) addressed the uncertainty concern and (3) added more discussion on the broader environmental implications and (4) provided our recommendations of mitigation measure. As a result, we believe we have significantly improved our paper. We appreciate the constructive feedback from both reviewers and welcome any further comments.

<u>Reviewer #2</u>:

The paper by Huang et al. "Estimating CO2 Emissions Due to Present and Future Suborbital Space Tourism Industry" discussed a hot debated topic after several high-profile suborbital launches in recent years. By using physics and thermodynamics principles, the authors developed an analytical framework with governing equations to estimate the space tourism industry's carbon footprint. I have several concerns about their methods and approximations.

The formula 3 doesn't hold outside of the troposphere. In stratosphere (20-50 km), temperature increases with altitude. Then in the mesosphere (50-100 km), temperature decreases again. But since the authors are studying the emission from launch to 100km, equation 3 only applies for certain segments.

Response: The reviewer is correct about the temperature profile and please allow us to clarify our approach as below. The lapse rate in Eq. 3 is only applicable within a certain altitude in the troposphere. For our analysis, Eq. 3 is used to only estimate air density up to 20 km and beyond that altitude air density is treated as zero, because the air density beyond 20 km is less than 2.74×10^{-4} kg/m^3, which means it is less than 0.02% of the air density at sea level. We stated that the air density was negligible after 20 km in our first version and in this revision, we have added more explanation from Lines 220 – 223 to make it clear how we use air density at different altitude in our calculation. Addition notes have been added to Eqns. 17 and 18.

Lines 220 - 223

R is the ideal gas constant, L is the temperature lapse rate (6.5 K/km), and h is the altitude. Eqn. 3~~1~~ is used to only calculate air density up to 20 km, above which the air density is treated as 0 because the air density beyond 20 km is less than 0.02% of the value at the sea level (53). ~~That also means the air drag will become zero after 20 km.~~

L28 can quantitively specify the numbers.

Response: The abstract has been revised to include more quantitative statements as suggested. These changes appear in Lines 30 – 38.

Lines 30 - 38
CO_2; however, it i's still associated with ~~a~~ 90 to 106 metric tons ~~significant amount~~ of indirect CO_2
is used as fuel, its emissions may become comparable with those using liquid hydrogen, but its maximum reduction in emission can only be about 10% because that is the amount associated with production. Generally, conventional rocket fuels generate more CO_2 than emerging fuels such as hydrogen and methane. Even though the total emissions of suborbital flights are still small compared with commercial aviation, their emission intensity (i.e., per passenger per hour) is enormous (83 - 217 tonst~~on~~ vs. 250 kg). If the demand for suborbital flights increases significantly,

There are grammar errors in the draft. Please proofread.

Response: We have proofread a few times to remove grammatical errors and improve wording and sentencing.

L169 Considering drag coefficient can change with rocket shape and speed, using a constant value is way too simplistic. The authors should at least identify the main stages during the trip, and use a customized drag coefficient for each, or replace C_d using a function of flight conditions at least.

Response: We appreciate the comment and would like to utilize this opportunity to clarify the difference between air drag coefficient and air drag. The air drag is the force depending on air drag coefficient, air density, velocity and frontal area, as shown in the equation below, which also the Eq. 2 in the paper.

$$F_{ad} = 0.5C_d\rho v^2 A$$

Air drag coefficient only depends on the shape of the spacecraft, which is usually determined by wind tunnel tests. The air drag coefficients of commonly seen shapes are provided below.

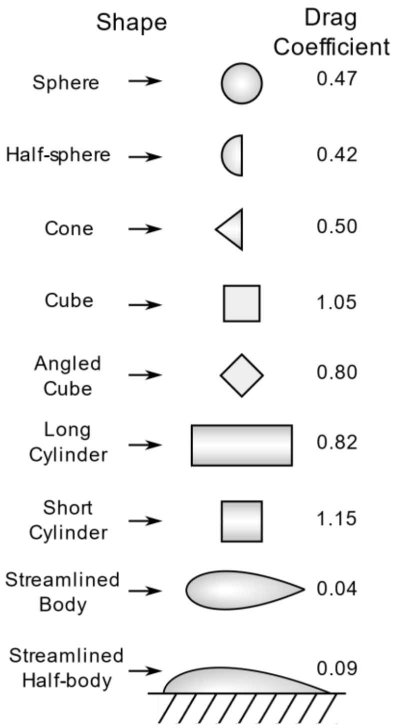

Measured Drag Coefficients

In summary, air drag depends on many factors, including air drag coefficient, air density, velocity and frontal area, while air drag coefficient only depends on shape.

According to the figure provided above, the air drag coefficient for half-sphere is 0.42 and for long cylinder is 0.82. Spacecraft for suborbital travel is typically a combination of half sphere and long cylinder; therefore, its air drag coefficient should be between 0.42 and 0.82. The NASA model test has verified that it is around 0.75, which is what NASA used for its design.

Table 2 it is interesting to see the exhaust velocity difference using liquid hydrogen and various oxidizers is all around 14%. But since the main fuel is the same (~hydrogen) in this case, oxidizers just play the role of oxidizers, so maybe that all makes sense. But what the authors did not do is for different fuels, the 14% factor might change.

Response: It is a good comment. To address it, we have spent a great deal of time finding the exhaust velocity difference between 1 atm and vacuum conditions for ***different*** fuels.

Based on the data we collected, the difference for other fuels is 14 - 15%. We have modified Table 2 to include new data, as shown below:

Table 2

	Fuels	H_2	CH_4	RP-1	HTPB
Exhaust velocity, v_e (m/s)	p_e at 1 atm	3816 - 4036	3127 - 3484	2941 - 3424	n/a
	p_e at vacuum	4461 - 4697	3692 - 4131	3510 - 4021	n/a
	Difference	14.3%	15.4%	15.0%	n/a

As a result, we have modified our formula to use 0.14 for liquid hydrogen and 0.15 for other fuels. The modification can be seen in a few formulas, such as Eqn. 12.

The change of 0.14 to 0.15 for CH_4, RP-1, and HTPB has a very limited impact on the calculation and has no impact on our overall conclusions.

Table 6 First need to list units on the table. I would also suggest the EF for hydrogen is highly uncertain while the authors assumed a linear trend and used a simple approximated value. At least some sensitivity analysis needs to be done to suggest the full range with uncertainty attached.

Response: We appreciate the suggestions. The emission factor is the ratio of emission mass to fuel mass. Therefore, it is unitless, i.e., g/g. We have added the explanation in the text and indicated that it is unitless in Table 6.

Table 6

	Direct emissions due to Combustion	Indirect emissions due to Production	
	Current and ~~/~~Future (g/g)	Current (mid-2020s) (g/g)	Future (2030s) (g/g)
Hydrogen	0	10.1~~10.7~~ – 11.8	~~7.15~~6.7 – 7.6
HTPB	2.2 – 2.6*	----	----
RP-1	3.16 - 3.5	0.5 - 2.1	0.4 - 1.54
CH_4	2.6 - 2.75	0.13 – 0.44~~0.27~~	0.1 - 0.18
Note: *the emission factor range for HTPB is calculated from its theoretical emissions from combustion and then considering engine efficiency.			

Regarding the variation of emission factors, we have conducted a detailed Monte Carlo simulation to cover a large possible variation range of emission factors so the uncertainty and variation of emissions can be presented. Now, the variations of emissions can be seen in Figures 4 and 7 and Table 7. Some examples are cited here for illustration purposes and the reviewer can find all the full revision in our revised paper.

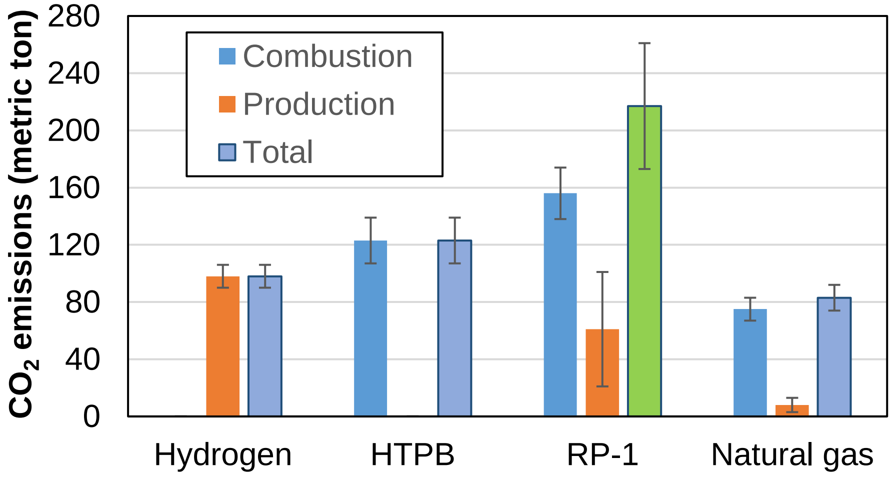

(new Figure 4(c))

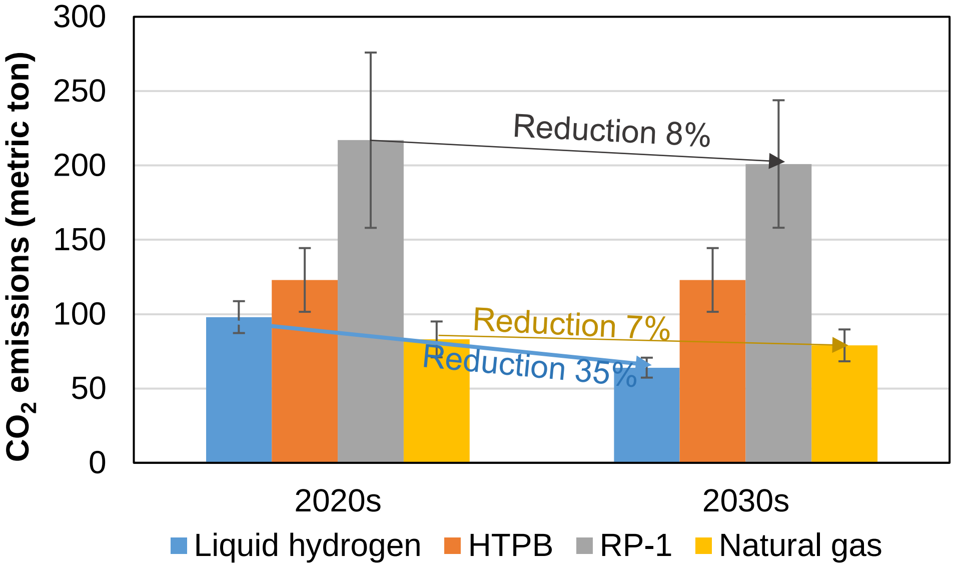

(new Figure 7)

L443 The authors can also try to dismiss the carbon footprint estimates for HTPB since the results will be quite uncertain already.

Response: We appreciate this suggestion and think the HTPB data also has value in this paper. We admit that the unavailability of a lot of information about HTPB, such as emissions associated with its ***production***, led to uncertainty to our estimation. However, the emissions from ***combustion*** alone would make it emit more than others. This information is valuable for decisions or policymaking in the future. We also have added some more discussion to warn readers of the uncertainty associated with HTPB estimation. We have added some sentences in Lines 771-773 to make the point clear.

Lines 771 - 773
For HTPB, CO_2 from combustion alone is already approximately 20% - 40% higher than those from production and combustion for liquid hydrogen and natural gas. Thus, HTPB should be considered a high-emission fuel.

L453 Before natural gas liquefaction, there can be flaring, combustion on sites for compression, these all lead to CO_2 emissions while the authors only considered

the liquefaction step. The use of 10% will be an underestimate and the authors need to find more data resources to constrain the upper bound.

Response: We appreciate this suggestion of these upstream emissions associated with natural gas production and have reviewed different studies worldwide and collected additional data on liquefaction-associated CO_2 emissions. Considering the world and U.S. data, we are now using 5 – 16% as the possible range of emissions due to CH_4 production. This range has been used in Monte Carlo simulation to estimate possible CO_2 emission ranges. This information can be found from Lines 483 – 496. As a result, Table 6 has been modified to reflect that change.

Lines 483 - 496

The CO_2 emissions associated with RP-1 fuel are estimated based on US EPA emission factor. The combustion of each gallon of kerosene would directly release 9.98 kg of CO_2 (100), which is converted to a mass ratio, i.e., emission factor. Koroneos, Dompros (101) and GHG Management Institute (95) indicated the emission factors associated with ~~0.051 kg/MJ for emissions associated with~~ kerosene production range from 0.5 to 2.1, which ~~is used here to quantify~~are listed in ~~the CO_2 emissions from kerosene production (~~Table 6~~)~~.

Detailed data on CO_2 emissions associated with natural gas production is not available, but Zhang, Cusworth (96) concluded that natural gas liquefaction released only 5 - 16% CO_2 relative to end-use combustion. Okamura, Furukawa and Tamura, Tanaka reported that 230 – 410 g of CO_2 is released to liquefy each kilogram of natural gas (102, 103), which is equivalent to 8.5% to 15.2% of end-use emissions. Howarth suggested that liquefaction accounts for 8.8% CO_2 emissions relative to end-use based on the U.S. data (104). Thus, it is supportive to use 5 – 16% as the lower and upper bound of CO_2 emissions due to liquefaction. ~~The average (10%) is used to estimate the CO_2 emission associated with production.~~ It is noteworthy that at this moment, SpaceX is using

L499 I doubt other columns will have similar range of uncertainty considering the large uncertainty in emission factors and other relevant variables. The other thing the authors should improve is to be more quantitative. They can simply add uncertainty ranges to all columns.

Response: As suggested, we have conducted Monte Carlo simulations to define the variation of calculated values. Thus, we have updated Table 7 and Figures 4 and 7 to include new data.
