# Supplementary material for: Estimating CO2 emissions due to present and future suborbital space tourism industry
Source: PLoS One. 2025 Aug 20;20(8):e0328456. doi: 10.1371/journal.pone.0328456 (PMC12367173; doi:10.1371/journal.pone.0328456)
Supplement: Attachment — Submitted filename: responses to review comments.docx [file pone.0328456.s004.docx]

**Responses to Review Comments**

(Manuscript No.: PONE-D-25-00871R1)

Angela M. Huang and Yangyang Xu*

(* corresponding author)

The authors would like to thank the editor and reviewer(s) for their careful review and valuable comments/suggestions. Below please find our point-by-point responses to the editor’s and reviewers’ comments and the changes we made to address the comments/suggestions. All the review comments/suggestions are presented in ***bold Italic Arial*** while our responses are presented in Normal Arial. **All the page numbers or line numbers cited herein are based on the version of the paper with tracked changes, which is submitted in this revision.**

**Editor’s comments**:

***I appreciate your efforts to address the majority of my inquiries. My final apprehension pertains to the disparity in air density at the 20 km mark. I discovered that the air density at 20 km is approximately 7% of the density at sea level, which is not the 0.02% that the authors had previously identified. Consequently, it is not negligible, particularly when studying high-speed flying objects. The authors must revise their segment calculations into at least three bins: 0-20, 20-60 (above 60 km, it is safe to assume density to be zero), and 60-100 km.***

Response: We have carefully addressed the concern and taken the Reviwer#2’s suggestion by considering the air drag from 20-60 km into our calculation. Please see our detailed response to Reviewer#2 for the changes we made.

**Reviewers' comments**:

**Comments to the Author**

***1. Is the manuscript technically sound, and do the data support the conclusions?

The manuscript must describe a technically sound piece of scientific research with data that supports the conclusions. Experiments must have been conducted rigorously, with appropriate controls, replication, and sample sizes. The conclusions must be drawn appropriately based on the data presented.***

***Reviewer #2: (no response)***

Response: We have considered the air drag from 20 – 60 km as suggested by the reviewer.

***2. Is the manuscript technically sound, and do the data support the conclusions?***

***Reviewer #2: Yes***

Response: n/a

***3. Has the statistical analysis been performed appropriately and rigorously?***

***Reviewer #2: Yes***

Response: n/a

***4. Have the authors made all data underlying the findings in their manuscript fully available?***

***Reviewer #2: Yes***

Response: n/a

***5. Is the manuscript presented in an intelligible fashion and written in standard English?
Reviewer #2: Yes***

Response: n/a

**Reviewer #2**:

***Thanks for addressing most of my comments. My last concern is on the air density difference on the 20 km mark. What I found out is air density at 20 km is about 7% of density on sea level, not the 0.02% the authors identified, so it is not negligible especially we are studying high speed flying object. The authors need to revise their segment calculations into at least 3 bins, 0-20, 20-60 (above 60 km, seems we can safely assume density to be zero), 60 -100 km.***

Response: We appreciate the reviewer’s comment that allows us to further improve our calculation. Based on the comments, we have made changes to account for the air drag in three flight segments, i.e., 0-20, 20-60 and 60-100 km. The added description is cited below:

**Lines 195-198**

Now the air drag equation is presented as piecewise function to considered air drags from 0 to 20km, from 20 to 60km and above 60km, as shown below:

**Eqn. 3**

All relevant equations have been modified to reflect this change, including the governing equation – Eqn. 17. This revision will only change our calculation slightly for two reasons. First, even though the air density at altitude from 20 – 60 km is not zero, it is very small compared with air density at sea level. Second, air density from 20 – 60 km leads to higher fuel consumption during launching but lower fuel consumption at landing because air drag facilitates landing. Therefore, the overall change in fuel consumption is less than 5%, as indicated in Table 7. Some of the numbers even do not change due to decimal rounding.

**Table 7**

The slight change in fuel consumption does not impact most of our conclusions except the following few as summarized below:

|  | Before this revision | After this revision |
| --- | --- | --- |
| Current emission of suborbital flight (ton) | 83 to 217 | 85 to 226 |
| Total project emission (Mt ton) | 20 | 21 |
| Comparison with other countries | Guatemala, which is 90^th^ in the world in terms of annual emission | Bolivia, which is 84^th^ in the world in terms of annual emission |

In summary, the slight change in the number does change the major conclusions we drew from this study.
